# A Cerebral Recovery Index (CRI) for early prognosis in patients after cardiac arrest

**DOI:** 10.1186/cc13078

**Published:** 2013-10-22

**Authors:** Marleen C Tjepkema-Cloostermans, Fokke B van Meulen, Gjerrit Meinsma, Michel JAM van Putten

**Affiliations:** 1 Clinical Neurophysiology, MIRA - Institute for Biomedical Technology and Technical Medicine, University of Twente, Enschede, The Netherlands; 2 Department of Neurology and Clinical Neurophysiology, Medisch Spectrum Twente, Enschede, The Netherlands; 3 Biomedical Signals and Systems, MIRA - Institute for Biomedical Technology and Technical Medicine, University of Twente, Enschede, The Netherlands; 4 Hybrid Systems, Centre for Telematics and Information Technology, University of Twente, Enschede, The Netherlands

## Abstract

**Introduction:**

Electroencephalogram (EEG) monitoring in patients treated with therapeutic hypothermia after cardiac arrest may assist in early outcome prediction. Quantitative EEG (qEEG) analysis can reduce the time needed to review long-term EEG and makes the analysis more objective. In this study, we evaluated the predictive value of qEEG analysis for neurologic outcome in postanoxic patients.

**Methods:**

In total, 109 patients admitted to the ICU for therapeutic hypothermia after cardiac arrest were included, divided over a training and a test set. Continuous EEG was recorded during the first 5 days or until ICU discharge. Neurologic outcomes were based on the best achieved Cerebral Performance Category (CPC) score within 6 months. Of the training set, 27 of 56 patients (48%) and 26 of 53 patients (49%) of the test set achieved good outcome (CPC 1 to 2). In all patients, a 5 minute epoch was selected each hour, and five qEEG features were extracted. We introduced the Cerebral Recovery Index (CRI), which combines these features into a single number.

**Results:**

At 24 hours after cardiac arrest, a CRI <0.29 was always associated with poor neurologic outcome, with a sensitivity of 0.55 (95% confidence interval (CI): 0.32 to 0.76) at a specificity of 1.00 (CI, 0.86 to 1.00) in the test set. This results in a positive predictive value (PPV) of 1.00 (CI, 0.73 to 1.00) and a negative predictive value (NPV) of 0.71 (CI, 0.53 to 0.85). At the same time, a CRI >0.69 predicted good outcome, with a sensitivity of 0.25 (CI, 0.10 to 0.14) at a specificity of 1.00 (CI, 0.85 to 1.00) in the test set, and a corresponding NPV of 1.00 (CI, 0.54 to 1.00) and a PPV of 0.55 (CI, 0.38 to 0.70).

**Conclusions:**

We introduced a combination of qEEG measures expressed in a single number, the CRI, which can assist in prediction of both poor and good outcomes in postanoxic patients, within 24 hours after cardiac arrest.

## Introduction

Early prognosis in patients with postanoxic encephalopathy after cardiac arrest is limited, especially due to treatment with mild hypothermia and sedation [1,2]. In only 34% to 60% of patients treated with hypothermia after cardiac arrest, will consciousness return [3-5]. Electroencephalography (EEG) monitoring may assist in early prognosis [6-9]. However, analysis of long-term EEG registrations is very time-consuming and can be done only by an experienced electroencephalographer [10-14]. Furthermore, visual EEG interpretation will always be partially subjective [11,14].

Quantitative EEG (qEEG) analysis can reduce the time needed to review long-term EEG, and makes the analysis more objective [12-14]. Additionally, qEEG analysis can be used to reveal and display trends in EEG patterns over longer time periods [13]. Thereby it can be used to study time constants of improvement in the EEG. In a cohort of 30 patients Wennervirta *et al*. [15] showed that individual qEEG features, such as the burst-suppression ratio, the response entropy, and the state entropy, differed between good- and poor-outcome groups during the first 24 hours after cardiac arrest [15]. A response entropy of ≤12.53 and a subband entropy of ≤11.84 at 24 hours after cardiac arrest both had a sensitivity of 78% and a specificity of 81% for predicting poor neurologic outcome [15]. These results are promising and could possibly be improved by using a combination of multiple qEEG features integrated as a single index.

In this study, we analyzed five qEEG features and combined these into the Cerebral Recovery Index (CRI), which provides a single number that can be used for prognostication in patients treated with mild hypothermia after cardiac arrest.

## Materials and methods

### Patients

From June 2010 to February 2013, we monitored all patients after cardiopulmonary resuscitation who were admitted to the ICU of our hospital (Medisch Spectrum Twente, Enschede, The Netherlands) for therapeutic hypothermia. A detailed description of patient-inclusion criteria was given in [8]. In short, all adult patients (aged >18 years) who were resuscitated after a cardiac arrest, remained comatose, and were admitted to the intensive care unit (ICU) to receive therapeutic hypothermia (at 33°C, maintained for 24 hours) were included. Patients with additional neurologic injuries were excluded. The data of the first patients (from June 2010 to July 2011), which we also used in our previous study on the evaluation of predictive value of visual analysis of the EEG [8], were used as training data to define qEEG features and optimize parameter settings. The EEG recordings of the patients included after July 2011 were used as test data, and therefore used only for evaluation. The Institutional Review Board of the Medisch Spectrum Twente waived the need for informed consent for EEG monitoring during ICU stay and for the follow-up after 3 and 6 months by telephone. However, for additional electrophysiological and clinical evaluation after discharge from the ICU in the first 60 patients, the local institutional review board approval and written informed consents were obtained.

### EEG recordings

EEG recordings were started as soon as possible after the patients’ arrival on the ICU and continued up to 5 days or until discharge from the ICU. For practical reasons, EEG recordings were not started late at night. Instead, for patients admitted to the ICU after 11 PM, the recordings were started the next morning at 7 AM. Twenty-one silver/silver chloride cup electrodes were placed on the scalp according to the international 10–20 system. Recordings were made by using a Neurocenter EEG recording system (Clinical Science Systems, Voorschoten, The Netherlands). All EEG analyses were performed offline. EEG data played no role in actual prognostication of outcome or treatment decisions. However, the treating physicians were not completely blinded to the EEG to allow treatment of epileptiform discharges. Treatment of epileptiform activity was left to the discretion of the treating physician. Generalized periodic discharges were also interpreted as epileptiform activity, and treated with antiepileptic drugs. However, no treatment protocol existed for treatment, because evidence for effect of treatment is lacking. Therefore, both the nature and the intensity of treatment differed among physicians. In general, only moderate levels of antiepileptic drugs were given, and treatment never reached an intensity to induce burst-suppression EEG; barbiturates were not used.

#### Selecting EEG epochs

EEG epochs of 5 minutes were automatically selected every hour during the first 48 hours after resuscitation and every 2 hours during the remainder of the registration. In this selection, the EEG epoch with the least number of artefacts was chosen, after applying an artefact-detection algorithm. In this algorithm, EEG data from 10 minutes before until 10 minutes after the selected time point were assessed. The EEG data of these 20 minutes were divided into 30-second segments. For each segment, a value for the amount of artefacts was determined by calculating the number of high-voltage peaks (movement artefacts), the power ratio between frequencies inside the EEG range and higher frequencies (muscle activity), and the number of channels that contains zeros (unstacked wires or loose electrodes). Finally, the 10 consecutive segments with the lowest summed artefact values were selected, resulting in a 5-minute epoch. In EEG registrations with too many artefacts during the complete 20 minutes, no epoch was selected for that selection moment.

#### Quantitative EEG features

First, all epochs were filtered by a zero-phase 6^th^ order Butterworth bandpass filter (0.5 to 30 Hz) and transformed to the source derivation. Subsequently, the qEEG analysis was performed. Five features were used: the power, the Shannon entropy, the alpha-to-delta ratio, the regularity (a feature we developed to distinguish burst-suppression patterns from continuous EEG patterns), and coherence in the delta band. These features were motivated by the criteria that a neurologist evaluates during visual analysis of an EEG. After calculation of the values of the five qEEG features, all features were normalized between 0 and 1 with a smooth exponential function, and combined into one overall score, the Cerebral Recovery Index (CRI).

All qEEG features, except the feature for regularity of the amplitude, were first calculated per EEG channel and per 10-second segments separately and subsequently averaged over time and over all channels. The regularity feature was calculated per channel for the complete 5 minutes at once, and then averaged over all EEG channels.

Power: To quantify the power of the EEG, the standard deviation (SD) of the EEG was calculated. As the mean of the signal can be expected to be negligibly small after filtering, the SD is equivalent to the mean power of the signal.

Shannon entropy: An analytic technique to quantify the irregularity of a stochastic signal is entropy. Overall, entropy describes the complexity or unpredictability of a signal. In this study, we used the Shannon entropy (H_Sh_), first defined by Shannon and Weaver as:

(1) $$H_{\mathrm{sh}}=-\sum_{i=1}^{N}p\left(x_{i}\right)\;\log_{2}p\left(x_{i}\right)\;$$

where *x*_*i*_ is the amplitude of the signal and *p(x*_*i*_) the probability of its occurrence in the signal segment [16,17]. The probability density function *p(x*_*i*_*)* was estimated by using the histogram method in which the amplitude range of the signal was linearly divided into bins (from -200 μV to 200 μV, with a bin width of 1 μV.)

Alpha-to-delta ratio: The alpha-to-delta ratio (ADR) [13,18-20] was calculated as the power ratio between the alpha (8 to 13 Hz) and delta frequency band (0.5 to 4 Hz). To calculate this power ratio, a power spectral density was estimated by using Welch’s averaged periodogram method by using a Hamming window with a length of 2 seconds, resulting in a spectral-density estimation with a resolution of 0.5 Hz.

**Figure 1:**
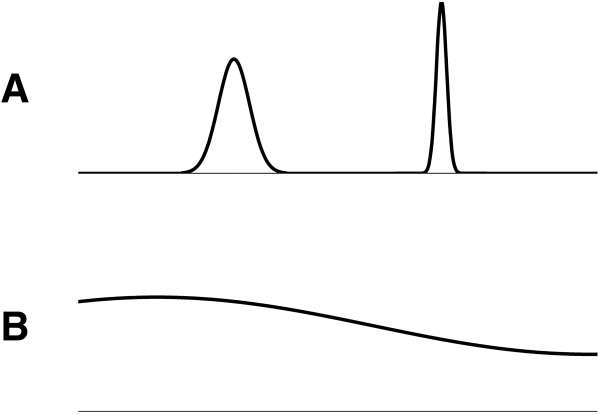
**Example of two signals with different variance in amplitude.** The signal in **(A)** shows two short periods with high amplitude on a zero background; the variance in amplitude in this signal is relatively high, whereas the signal in **(B)** has a more-regular or constant amplitude. The signal in **A** can be compared with an EEG showing a burst-suppression pattern, whereas the signal in **B** can be compared with an EEG with continuous amplitude. This is expressed in the regularity index (compare Equation 2 and Figure 2).

**Figure 2:**
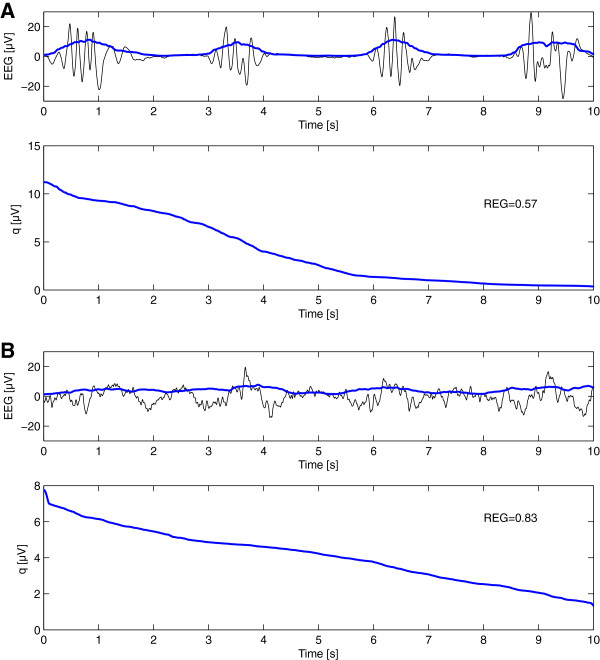
**Calculating the regularity of the amplitude (REG) in an EEG showing a burst-suppression pattern (A) and a diffusely slowed pattern (B).** In the top graphs, the raw EEG is shown (black), together with the EEG, after squaring and applying a moving average filter (with a window of 0.5 seconds) (blue). In the bottom graphs, the signal *q* is obtained after sorting this smoothed signal in descending order. The calculated value for the regularity (REG) is the normalized variance of this sorted signal *q* (compare Equation 2). REG is normalized from 0 to 1, where a higher value corresponds to a signal with a more regular amplitude, as illustrated.

Regularity: To separate burst-suppression patterns from continuous EEG patterns (with a regular, constant amplitude), we developed a feature to evaluate the regularity of the amplitude of a signal. In Figure 1, we present two signals as an example. Figure 1A shows a signal with a high variance in amplitude, and Figure 1B, a signal with more regular amplitude. In this technique, we first squared the signal and applied a moving-average filter with a window of 0.5 seconds to create a nonnegative smooth signal. The window length of the moving average was set at 0.5 seconds. A longer window would average out the differences in activity between subsequent bursts and suppressions, whereas a shorter window length would not average out the individual peaks within one burst. Subsequently, we sorted the values of the smoothed signal in “descending” order (see Figure 2). The normalized standard deviation of this sorted signal was then calculated as a feature for regularity (REG) in amplitude of the data:

(2) $$\mathrm{REG}=\sqrt{\frac{{\sum \sum }_{i=1}^{N}i^{2}q\left(i\right)}{\frac{1}{3}N^{2}{\sum \sum }_{i=1}^{N}q\left(i\right)}\;}$$

with *N* the length of the signal in samples and *q* the sorted signal. The nominator calculates the standard deviation of the sorted signal, which is normalized in a range between 0 and 1 by the denominator. The REG value of a signal with constant amplitude is 1, independent of the amplitude of the signal. A signal with relatively low amplitude (suppression) that contains a short period of higher amplitude (burst) will have a value close to zero; if there are more or longer bursts, the REG value will increase. Two examples of this technique applied on EEG data showing a burst-suppression pattern and a normal EEG pattern are given in Figure 2A and B, respectively. Note that the REG value for the burst-suppression EEG (Figure 2A) is lower than of the normal continuous EEG (Figure 2B), indicating that the burst-suppression EEG shows more spread in amplitude.

Coherence in the delta band: To quantify EEG patterns with an abnormal high synchronization level, the mean coherence (COH) in the delta band (0.5 to 4 Hz) between all possible combinations of EEG channels was implemented. In the calculation of the coherence, we used a Hanning window with a length of 4 seconds and an overlap of 2 seconds.

#### Feature combination

**Figure 3:**
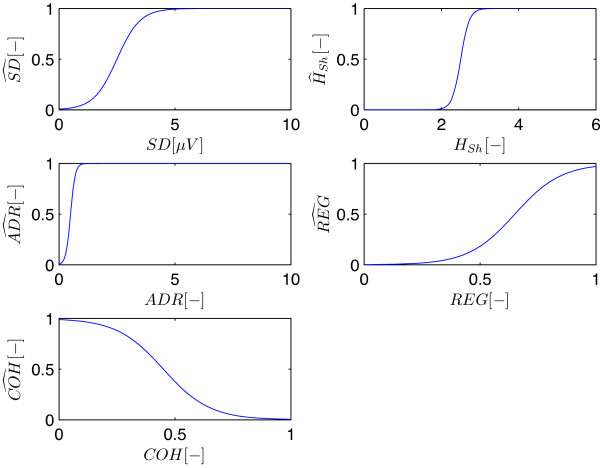
**Normalized qEEG scores.** All five qEEG values are normalized using a smooth sigmoid function (Equations 3, 4, 5, 6, 7), resulting in score for each feature (annotated with a hat) between 0 and 1. (SD = standard deviation, H_Sh_ = Shannon entropy, ADR = alpha to delta ratio, REG = regularity, COH = coherence).

Finally, the five qEEG features were combined into a single number, the Cerebral Recovery Index (CRI). First the value of each qEEG feature was normalized in the range from 0 to 1, with 0 corresponding to a pathologic EEG and 1 corresponding to a physiologic EEG. These normalized qEEG scores (annotated with a hat) are schematically displayed in Figure 3 and expressed as:

(3) $$\hat{\mathrm{SD}}=1/\left(1+e^{-2\left(\mathrm{SD}-2.5\right)}\right)\;$$

(4) $$\hat{H_{\mathrm{Sh}}}=1/\left(1+e^{-9\left(H_{\mathrm{Sh}}-2.5\right)}\right)$$

(5) $$\hat{\mathrm{ADR}}=1/\left(1+e^{-10\left(\mathrm{ADR}-0.5\right)}\right)$$

(6) $$\hat{\mathrm{REG}}=1/\left(1+e^{-10\left(\mathrm{REG}-0.65\right)}\right)$$

and

(7) $$\hat{\mathrm{COH}}=1/\left(1+e^{10\left(\mathrm{COH}-0.45\right)}\right)$$

The values for the parameters in these expressions were set after visual inspection of the data of the training set. We did this for each feature independently, selecting the data that was most relevant for that specific feature. For example, for the REG feature we compared burst-suppression EEGs with normal EEGs showing continuous activity, while for the SD feature we compared iso-electric and low-amplitude EEGs with continuous EEGs.

As the power of an EEG signal is a requirement for a normal EEG - if there is no power at all, the EEG is flat and all other features are useless - in the combined score, ($\hat{\mathrm{SD}}$) was multiplied with the mean of the other four qEEG scores. However due to the sigmoid shape of the curve for $\hat{\mathrm{SD}}$ (Equation 3, Figure 3), the value of the CRI is independent for further changes in power once the power has reached a certain minimal threshold; above a mean amplitude of 5 μV the value of the $\hat{\mathrm{SD}}$ goes to 1. The resulting expression for the CRI is:

(8) $$\mathrm{CRI}=\hat{\mathrm{SD}}\;\left(\frac{\hat{H_{\mathrm{Sh}}}+\hat{\mathrm{ADR}}+\hat{\mathrm{REG}}+\hat{\mathrm{COH}}}{4}\right)$$

To evaluate the time dependency of the CRI, we introduce a “recovery function”, *R(t)*, expressed as:

(9) $$R\left(t\right)=a_{0}+a_{1}H\left(t-\delta \right)\left(1-e^{-\left(t-\delta \right)/\tau }\right)$$

with *H* the Heaviside or step function. The constants *a*_*0*_ and *a*_*1*_, delay δ, and time constant *τ* were estimated by using the median values of the CRI, for patients with both good and poor neurologic outcomes.

### Outcome assessment

Neurologic outcome assessment was performed at 3 and 6 months after cardiac arrest during a personal meeting or based on a telephone call, and was always performed by the same author (MT-C). The primary outcome measure was the best score within 6 months on the 5-point Glasgow-Pittsburgh CPC scores [21]. Outcome was dichotomized between “good” and “poor.” A good outcome was defined as a CPC score of 1 or 2 (no or moderate neurologic disability), and a poor outcome, as a CPC score of 3, 4, or 5 (severe disability, coma, or death).

### Statistical analysis

Collected baseline characteristics include age, sex, weight, location of cardiac arrest (in-hospital versus out-of-hospital), cause of cardiac arrest, and initial cardiac rhythm. Information about the administered sedative (propofol and midazolam) and analgesic (fentanyl and remifentanyl) drugs and their maximum dose within the first 24 hours were collected. Statistical analysis for the variables that were categoric was performed by using a Pearson χ^2^ test when no subgroup had an expected count less than 5, or else a Fisher Exact test was performed. For continuous variables, an independent *t* test was applied after confirming that these variables were normally distributed.

At 12, 18, 24, and 36 hours after cardiac arrest, we determined the area under the curve (AUC) of the receiver operating characteristic (ROC) curve. Furthermore, we defined at each of these time points two thresholds for the CRI score, one corresponding to a 100% specificity for predicting poor neurologic outcome, and one corresponding to a 100% specificity for predicting good neurologic outcome. For each threshold, we calculated the sensitivity, specificity, positive predictive value (PPV), and negative predictive value (NPV), and their 95% confidence intervals (CIs).

## Results

In total, 109 consecutive patients were included in the study. The first 56 patients were used as the training set, and the remaining 53 patients were included in the test set. In the training set, 27 (48%) of the 56 patients had good neurologic outcome (best CPC score ≤2 within 6 months). In the test set, 26 (49%) of the 53 patients had good neurologic outcome. Additional patient information of the training set is given in [8]. Table 1 summarizes the patient characteristics of the test set. Both in the training and test-set groups, patients with good neurologic outcome and patients with poor neurologic outcome were sedated at the same dosage levels. However, in the test group, patients with good neurologic outcome received a slightly higher dose of propofol in comparison to patients with poor neurologic outcome (Table 1).

**Table 1:** Comparison of patient characteristics between the patients with good neurologic outcome and poor neurologic outcome in the test set

	**Poor neurologic outcome (CPC 3–5)**	**Good neurologic outcome (CPC 1–2)**	***P *** **value**
Number of patients	27	26	-
Number of male	19 (70%)	20 (77%)	0.59
Age (years)	63 (STD 13)	58 (STD 11)	0.14
	(range, 27 to 82)	(range, 35 to 79)	
Number of OHCA	23 (85%)	23 (89%)	1.00
Initial rhythm:			
VF	8 (30%)	23 (89%)	0.00
Asystole	14 (52%)	0 (0)	
Bradycardia	1 (4%)	0 (0)	
Unknown	4 (15%)	3 (12%)	
Presumed cause of CA:			
Cardiac	17 (63%)	17 (65%)	0.57
Other origin	6 (22%)	3 (12%)	
Unk*n*own	4 (15%)	6 (23%)	
Patients sedated with propofol	27 (100%)	26 (100%)	-
Propofol dose (mg/h/kg)	2.8 (STD 1.0)	3.4 (STD 1.0)	0.03
	(range, 0.9 to 4.8)	(range: 1.3 to 5.4)	
Patients sedated with midazolam	8 (30%)	6 (23%)	0.59
Midazolam dose (μg/kg/hr)	80 (STD 65)	73 (std 35)	0.84
	(range, 30 to 214)	(range, 33 to 125)	
Patients treated with fentanyl	18 (67%)	19 (73%)	0.61
Fentanyl dose (μg/h/kg)	1.5 (STD 0.8)	1.9 (STD 0.7)	0.13
	(range, 0.6 to 3.6)	(range, 0.9 to 2.7)	
Patients treated with remifentanil	11 (41%)	7 (27%)	0.29
Remifentanil dose (μg/h/kg)	4.0 (STD 2.6)	5.5 (STD 3.0)	0.28
	(range, 1.0 to 7.0)	(range. 3 to 11)	

Medication doses are given as the maximum drug dose during the first 24 hours. CPC, Cerebral Performance Category; OHCA, out-of-hospital cardiac arrest; VF, ventricular fibrillation; CA, cardiac arrest.

**Figure 4:**
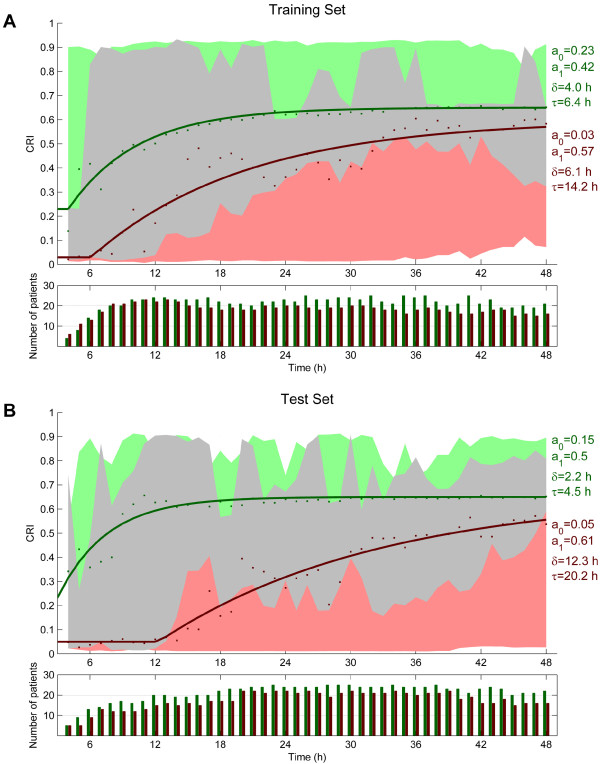
**Values of the Cerebral Recovery Index (CRI) for the training (A) and test (B) sets.** The green and red dots are the median values for patients with good and poor neurologic outcome at each time point; the green and red areas are the corresponding ranges. The grey represents the area where the green and red areas overlap. The fitted recovery functions, *R(t)* (Equation 9), are given as a solid line. Note that the largest difference between the fitted CRI curves is present between 12 and 24 hours after cardiac arrest.

Figure 4A and B shows the median CRI values of patients with good and poor neurologic outcomes and their corresponding ranges. Figure 4A shows the results of the training set, and Figure 4B, the test set. Both the training and test-set patients with good neurologic outcome have an overall higher CRI than the group of patients with poor neurologic outcome. We obtained a reasonable fit of the mean CRI values by using the recovery function given by Equation 9. Note that the largest difference between the fitted recovery curves is present between 6 and 24 hours after cardiac arrest. The time constant is substantially larger in the patients with poor neurologic outcome (τ = 14.2 hours in the training set and τ = 20.2 hours in the test set) in comparison to the patients with good neurologic outcome (τ = 6.4 in the training set and τ = 4.5 hours in the test set), indicating that the EEGs of patients with good neurologic outcome show a faster improvement.

Tables 2A and B show the results for predicting poor outcome at 12, 18, 24, and 36 hours after cardiac arrest. Table 2A shows the results for the training set, and Table 2B, for the test set. At 18 or 24 hours, the CRI performs best. At 24 hours after cardiac arrest, a CRI ≤ 0.29 was always associated with poor neurologic outcome, with a sensitivity 0.55 (CI, 0.32 to 0.76) at a specificity of 1.00 (CI, 0.86 to 1.00) in the test set. This results in a PPV of 1.00 (CI, 0.73 to 1.00) and a NPV of 0.71 (CI, 0.53–0.85). At the same time point, a CRI >0.69 can be used for predicting good outcome, with a sensitivity of 0.25 (CI, 0.10 to 0.14) at a specificity of 1.00 (CI, 0.85 to 1.00) in the test set, and a corresponding NPV of 1.00 (CI, 0.54 to 1.00) and a PPV of 0.55 (CI, 0.38 to 0.70).

**Table 2:** Sensitivity, specificity, positive predictive value (PPV), negative predictive value (NPV), and area under the receiver operating characteristic curve (AUC) for predicting neurologic outcome in the training set (A) and test set (B) at different time points after cardiac arrest

**Time**	**AUC**	**CRI**	**Predicting**	**Sensitivity (CI)**	**Specificity (CI)**	**PPV (CI)**	**NPV (CI)**
**A. Training set**							
12 h	0.83	<0.04	Poor outcome	0.27 (0.11–0.50)	1.00 (0.86–1.00)	1.00 (0.54–1.00)	0.60 (0.43–0.75)
		>0.90	Good outcome	0.13 (0.03–0.32)	1.00 (0.85–1.00)	1.00 (0.29–1.00)	0.51 (0.35–0.67)
18 h	0.69	<0.19	Poor outcome	0.28 (0.10–0.53)	1.00 (0.85–1.00)	1.00 (0.48–1.00)	0.63 (0.45–0.79)
		>0.91	Good outcome	0.05 (0.00–0.22)	1.00 (0.81–1.00)	1.00 (-)	0.46 (0.30–0.63)
24 h	0.87	<0.35	Poor outcome	0.45 (0.23–0.68)	1.00 (0.85–1.00)	1.00 (0.66–1.00)	0.68 (0.49–0.83)
		>0.61	Good outcome	0.57 (0.35–0.77)	1.00 (0.83–1.00)	1.00 (0.75–1.00)	0.67 (0.47–0.83)
36 h	0.74	<0.32	Poor outcome	0.28 (0.10–0.53)	1.00 (0.86–1.00)	1.00 (0.48–1.00)	0.65 (0.75–1.00)
		>0.91	Good outcome	0.04 (0.00–0.21)	1.00 (0.81–1.00)	1.00 (-)	0.44 (0.28–0.60)
**B. Test set**							
12 h	0.74	<0.02	Poor outcome	0.13 (0.02–0.40)	1.00 (0.83–1.00)	1.00 (0.16–1.00)	0.60 (0.42–0.77)
		>1.00	Good outcome	0.00 (0.00–0.17)	1.00 (0.78–1.00)	-	0.43 (0.26–0.60)
18 h	0.94	<0.18	Poor outcome	0.59 (0.33–0.82)	1.00 (0.85–1.00)	1.00 (0.69–1.00)	0.76 (0.56–0.90)
		>0.57	Good outcome	0.64 (0.41–0.83)	1.00 (0.80–1.00)	1.00 (0.77–1.00)	0.68 (0.46–0.85)
24 h	0.87	<0.29	Poor outcome	0.55 (0.32–0.76)	1.00 (0.86–1.00)	1.00 (0.73–1.00)	0.71 (0.53–0.85)
		>0.69	Good outcome	0.25 (0.10–0.47)	1.00 (0.85–1.00)	1.00 (0.54–1.00)	0.55 (0.38–0.70)
36 h	0.84	<0.22	Poor outcome	0.30 (0.12–0.54)	1.00 (0.86–1.00)	1.00 (0.54–1.00)	0.63 (0.46–0.78)
		>1.00	Good outcome	0.00 (0.00–0.14)	1.00 (0.83–1.00)	-	0.45 (0.30–0.61)

At each time point, we selected two thresholds for the Cerebral Recovery Index (CRI), one corresponding to a 100% specificity for predicting poor neurologic outcome, and one corresponding to a 100% specificity for predicting good neurologic outcome. In addition, the 95% confidence intervals (CIs) are given.

The threshold of the CRI of >1.00 means that no threshold could be chosen in which good neurologic outcome was predicted correctly in any of the patients without having any false positives, resulting in a sensitivity of 0.

## Discussion

Growing evidence suggests that EEG monitoring can play a significant role in the prediction of neurologic outcome in patients treated with hypothermia after cardiac arrest [6-9]. In addition to prognostic parameters based on visual interpretation of the EEG, we introduce the Cerebral Recovery Index (CRI), based on five qEEG features that grade the EEG patterns as observed in patients after cardiac arrest. This index may assist in the prediction of neurologic outcome after cardiac arrest. The advantage of a combined qEEG feature is that it is very simple to use, and trends in long-term EEG recordings can easily be studied, and it still covers more than one aspect of the EEG. We evaluated the CRI in a training group of 56 patients and a test group of 53 patients treated with hypothermia at the ICU after cardiac arrest.

Although many features can be extracted from EEG data [11,13,18,22], only five were used in this study. The selection of features was motivated by the EEG characteristics that neurophysiologists evaluate in visual interpretation of the EEG in patients after cardiac arrest. Subsequently, the features were combined into a single number: the Cerebral Recovery Index (CRI). For a proper evaluation of the CRI, we used independent training and test sets.

CRI scores are higher in patients with good outcome in comparison with patients with poor outcome and can be used to divide patients into three groups. The first group (green area in Figure 4) includes only patients with good neurologic outcome: at 24 hours after cardiac arrest; 25% of the patients with good neurologic outcome are in this group. The second group (red area in Figure 4) includes only patients with poor neurologic outcome, at 24 hours after cardiac arrest; this group includes around 55% of all patients with poor neurologic outcome. The last group (the grey area in Figure 4) includes patients with good as well as with poor neurologic outcomes. The first and second groups are of the most interest, because outcome prediction is 100% reliable in these patients.

The median values of the CRI of both groups of patients increased over time. However, the time constant in the recovery function *R(t)* of patients with good neurologic outcome is much smaller than in patients with poor neurologic outcome. This implies that the EEGs of patients with good neurologic outcome improve faster than do those of patients with poor outcome. We also showed that the CRI at 18 and 24 hours after cardiac arrest has a higher prognostic value in comparison to the values at 12 or 36 hours after cardiac arrest. This is similar to the time course reported in our previous study using visual analyses [8]. Therefore, it is important to start the EEG registration within the first 24 hours after cardiac arrest for maximal diagnostic yield. The CRI threshold for the prediction of poor outcome with a 100% specificity increases from a value of 0.02 to 0.29 in the period 12-24 hours. This reflects the evolution in EEG patterns, in agreement with visual inspection. For instance, an isoelectric EEG in the first hours after cardiac arrest is observed in patients with both a good and a poor outcome [6,8]. Such an isoelectric EEG will have a very low CRI score of almost zero, because the feature for the amplitude is multiplied with the summed values of the other four features. In all patients with good neurologic outcome, isoelectric EEG patterns, if initially present, will evolve within 24 hours to a burst-suppression or a continuous EEG pattern [8]. This is reflected by a CRI score >0.69 at 24 h. The interpretation of the EEG for prognostication, either quantitative with the CRI or with visual interpretation, must, therefore, be related to the time since cardiac arrest. We used 5-minute epochs of EEG with the fewest artefacts every hour or every 2 hours to limit the influence of artefacts on the CRI score. As the EEG patterns of patients after cardiac arrest in general evolve over hours [8], this interval is sufficient to track relevant changes.

The thresholds for the CRI slightly varied between the training and the test sets. For predicting poor outcome at 24 hours, the threshold decreased from 0.35 to 0.29, whereas for predicting good outcome at 24 hours, the threshold increased from 0.61 to 0.69. A larger test set is necessary to evaluate the thresholds of the CRI before application in the clinical setting.

Additional improvement might be the reduction of the irregularity in the border between the grey and green areas (representing a 100% specificity for predicting good outcome) in Figure 4. Because changes in the EEG typically occur slowly and continuously over time, this border should be smoother. The peaks in the border between the green and grey areas are therefore nonphysiological. At some points in time, the green and grey areas even completely overlap. This was caused by high-amplitude and high-frequency muscle artefacts, resulting in erroneously high CRI values in some patients with poor outcome, illustrating that in some patients, our automated selection of artifact-free EEG epochs was not sufficiently accurate.

Our method is completely automated, including the selection of artefact-free data. However, the automatic selection of artefact-free data is not perfect. An expert is needed to verify that the selected EEG epoch is indeed artefact free to assure that the CRI value is reliable. Therefore, quantitative EEG analysis can reduce the time needed to review long-term EEG and make interpretation more objective. However, it is primarily aimed to assist in the interpretation instead of replacing the visual analysis of the EEG by an expert neurologist.

The EEG registrations were accessible for the treating physicians at the ICU to allow treatment of epileptiform discharges. This could potentially have influenced decision making. However, the local protocols about patient treatments were strictly followed. As presently, the EEG of the first 24 hours is not included in the Dutch guidelines, these findings were never used in the decision making. An absent SSEP during normothermia was a reason to stop treatment, according to current guidelines. Other findings to stop treatment included absence of both pupillary light and cornea reflexes at day 3 after cardiac arrest, or an isoelectric or low-voltage EEG at day 3. In patients with a motor score >4, or in patients that showed clinical improvement, treatment was never stopped. The CRI values were calculated offline after inclusion of all patients, and were therefore not available for the treating physicians. The likelihood of a self-fulfilling prophecy is thus very small. Also, the dichotomization of continuous variables by using a threshold has its limitations [23]. A larger test set is necessary to evaluate the thresholds of the CRI before application in a clinical setting. Evaluation in a larger population may also result in change of thresholds, which could make it less suitable for decisions that require 100% accuracy. In clinical practice, therefore, in the interpretation of the CRI, the difference of the index from threshold should also be taken into account.

Another limitation might be that all patients were sedated during the hypothermic phase with propofol and, in some cases, additionally with midazolam in a low dose, which could have influenced the EEG registrations. However, both in this and in our previous study [8], we showed that at group level, patients with good neurologic outcome and patients with poor neurologic outcome were sedated at the same dosage levels. In the test group described in this study, patients with good neurologic outcome even received a slightly higher dose of propofol in comparison to patients with poor neurologic outcome. Although propofol may have a neuroprotective effect, this has only been shown in *in vitro* and *in vivo* established experimental models of acute cerebral ischemia [24,25]. No clinical data exist that establish neuroprotection by propofol in humans [26-28]. In our study, the mean difference in propofol dosage between the group of poor and good neurologic outcome is small. The main reason for the difference in propofol dosage used is probably that the postanoxic encephalopathy in patients with good neurologic outcome was less severe, resulting in more muscle activity. Therefore, a higher dosage of propofol was needed to limit shivering. This might indicate that the temperature regulation is less affected in patients with good neurologic outcome [29]. Furthermore, the improvements in EEG patterns were already visible within the first 24 hours after cardiac arrest, while patients were still treated with hypothermia and received sedative drugs. Therefore, it is very unlikely that the changes in EEG can be explained by the use of sedative drugs.

## Conclusions

We introduce the CRI to quantify and grade continuous EEG data of patients after cardiac arrest. The CRI can assist in prediction of both poor and good neurologic outcome within 24 hours after cardiac arrest.

## Key messages

• EEG monitoring in patients treated with therapeutic hypothermia after cardiac arrest may assist in early outcome prediction.

• Quantitative EEG analysis can reduce the time needed to review long-term EEG and makes the analysis more objective.

• We introduced a combination of five qEEG measures expressed in a single number, the Cerebral Recovery Index (CRI), which can assist in prediction of both poor and good outcome in postanoxic patients, within 24 hours after cardiac arrest.

• EEGs of patients with good neurologic outcome improve faster than those of patients with poor outcome, and the predictive value of the EEG is the highest in the window from 12 to 24 hours after cardiac arrest. Therefore, it is important to start the EEG registration within the first 24 hours after cardiac arrest for maximal diagnostic yield.

## Abbreviations

ADR: Alpha-to-delta ratio; CI: Confidence interval; COH: Coherence in the delta band; CPC: Cerebral performance category; CRI: Cerebral recovery index; EEG: Electroencephalogram; HSh: Shannon entropy; ICU: Intensive care unit; NPV: Negative predictive value; PPV: Positive predictive value; qEEG: quantitative electroencephalography; R: Recovery function; REG: Regularity; SD: Power calculated as the standard deviation of the signal.

## Competing interests

This work was financially supported by the Dutch Ministry of Economic Affairs, Agriculture and Innovation, province Overijssel, and province Gelderland through the ViP Brain Networks project. The funders had no role in study design, data collection and analysis, decision to publish, or preparation of the manuscript. The authors report no disclosures.

## Authors’ contributions

MT-C was responsible for the study design and conceptualization, performed the data interpretation and analysis, and drafted the manuscript. FvM was responsible for the study design and conceptualization, performed the data interpretation and analysis, and revised the manuscript. GM participated in the data interpretation and analysis, and revised the manuscript. MvP was responsible for the study design and conceptualization, participated in the data interpretation and analysis, and revised the manuscript. All authors read and approved the final manuscript.
